# Complete Genome Sequences of *Curtobacterium*, *Pantoea*, *Erwinia*, and Two Pseudomonas sp. Strains, Isolated from Apple Flower Stigmas from Connecticut, USA

**DOI:** 10.1128/MRA.00154-21

**Published:** 2021-05-13

**Authors:** Zhouqi Cui, Blaire Steven, Quan Zeng

**Affiliations:** a Department of Plant Pathology and Ecology, The Connecticut Agricultural Experiment Station, New Haven, Connecticut, USA; b Department of Environmental Sciences, The Connecticut Agricultural Experiment Station, New Haven, Connecticut, USA; University of Arizona

## Abstract

The genome sequences of 5 bacterial strains isolated from apple flower stigmas are reported. The strains represent species of *Curtobacterium*, *Pantoea*, and *Erwinia* and two species of Pseudomonas. These data will provide information for future taxonomic studies and information for investigating the metabolic and functional characteristics of apple flower-colonizing bacteria.

## ANNOUNCEMENT

The five bacterial strains, *Curtobacterium* sp. 24E2, *Erwinia* sp. 18B1, *Pantoea* sp. 1B4, Pseudomonas sp. 15A4, and Pseudomonas sp. 1079, were isolated from apple flower stigma samples collected on the apple cultivar ‘Early Macoun’ (Malus x domestica NY75414-1) planted at Lockwood Farm in Hamden, CT (41.406°N, 72.906°W) (1). Our previous data showed that *Enterobacteriaceae* and *Pseudomonadaceae* are the two predominant families of bacteria on apple stigma (2, 3). Within the *Enterobacteriaceae*, the genera *Pantoea* and *Erwinia* were predominant, and within the *Pseudomonadaceae*, Pseudomonas was the principal genus (3). The genus *Curtobacterium*, belonging to the phylum *Actinobacteria*, was much less abundant in the microbiome and was investigated as a representative of the apple stigma “rare biosphere” (3). These four strains were identified in our previous study by 16S rRNA gene comparisons (1). Acquisition of the full-genome sequence information of these strains will advance future investigations concerning the metabolic and functional requirements of the flower microbiome.

The stigma portion of an apple flower was dissected and immersed into 200 μl of 0.5× phosphate-buffered saline (PBS) in a sterile 1.5-ml microcentrifuge tube. The stigma sample was then sonicated for 5 min, followed by vortexing for 30 s. Then, 5 ml of PBS was spread onto a lysogeny broth (LB) agar plate to collect stigma resident bacteria (1). A single colony of each strain was inoculated into LB broth and incubated at 28°C overnight with shaking. Genomic DNA was extracted using the E.Z.N.A. (Omega, GA, USA) bacterial DNA kit according to the manufacturer’s instructions. The quality and quantity of isolated DNA were determined using the high-sensitivity D5000 ScreenTape system (Agilent Technologies, Santa Clara, CA) and the QuBit double-stranded DNA (dsDNA) broad-range assay (Thermo Fisher Scientific, Waltham, MA), respectively. For Nanopore sequencing, the ligation sequencing kit (SQK-LSK109; Oxford Nanopore Technologies) was used with ∼500 ng of input DNA for library construction. The library for Pseudomonas sp. 1079 was sequenced with a Flongle adaptor, and the other four strains were loaded together into an R9.4 flow cell with specific barcodes on the Oxford Nanopore MinION device. All base calling was performed with the “high-accuracy” model as integrated in MInKNOW v3.1.13 software.

Quality control of raw reads was performed on the raw sequencing data using LongQC (4). Genome assembly was performed using Flye, which is a long-read *de novo* genome assembly pipeline (5). Polishing was performed using the Burrows-Wheeler Aligner (BWA) v0.7.17 (6) and Racon v1.4.19 (7) with parameters specific for Nanopore read alignment and mapping. The polished output was further processed with Medaka v1.0.3 (https://github.com/nanoporetech/medaka). Quality assessment was determined with QUAST (8). The annotation was performed with the NCBI Prokaryotic Genome Annotation Pipeline v4.12 (PGAP) (9), with completeness and contamination checked using CheckM (10). The genome information of these five strains is listed in Table 1. The closest phylogenetic neighbors of these strains were identified by whole-genome average nucleotide identity (ANI) using FastANI v1.32 and 16S rRNA gene homology using blastn with the NCBI database. Default parameters were used for all software unless otherwise specified.

**TABLE 1:** Characteristics and accession numbers for genomes of the apple stigma bacterial isolates

Characteristic	Data for:				
	*Curtobacterium* sp. 24E2	*Erwinia* sp. 18B1	*Pantoea* sp. 1B4	Pseudomonas sp. 15A4	Pseudomonas sp. 1079
Mean raw sequence length (bp)	7,304	6,911	6,430	7,384	6,627
Total sequences (Gb)	2.17	2.05	2.10	1.37	0.57
Mean coverage (×)	555	384	391	229	91
Flow cell barcode sequences	TCCATTCCCTCCGATAGATGAAAC	ACGTAACTTGGTTTGTTCCCTGAA	AAGGATTCATTCCCACGGTAACAC	TAGGGAAACACGATAGAATCCGAA	^*a*^
Total sequence length (bp)	3,805,409	5,153,201	5,091,746	5,702,273	5,898,439
No. of scaffolds	1	5	6	1	2
Raw read *N*_50_ (bp)	15,616	13,014	12,702	15,569	14,902
Assembly *N*_50_ (bp)	3,805,409	4,898,912	4,115,195	5,702,273	5,894,865
GC content (%)	70.7	56.4	55.1	60.5	60.2
No. of genes	3,743	5,039	4,823	5,039	5,343
No. of protein-coding genes	3,043	3,661	3,711	4,318	4,389
No. of 16S rRNAs	4	7	7	5	6
No. of tRNAs	47	81	77	64	68
Completeness (%)^*b*^	87.33	87.18	96.23	98.57	97.27
Contamination (%)^*b*^	5.08	6.90	0.45	0.03	0.27
Closest relative (ANI [%], 16S rRNA gene identity [%])	Curtobacterium citreum (85.5, 90.75)	Erwinia aphidicola (98.7, 95.00)	Pantoea agglomerans (98.3, 99.87)	Pseudomonas graminis (91.7, 99.93)	Pseudomonas carnis (98.5, NA^*c*^)
Genome GenBank accession no.	CP068987	JAEUXA000000000	JAEUWZ000000000	CP068986	JAEUXB000000000
Raw read SRA no.	SRR13499755–SRR13499826	SRR13499599–SRR13499670	SRR13499518–SRR13499596	SRR13500215–SRR13500259	SRR13499671–SRR13499754

a This isolate was sequenced on a Flongle flow cell, while the other strains were barcoded and sequenced on an R9.4 flow cell.

b Based on marker gene analysis in the CheckM software package.

c NA, no available 16S rRNA gene sequence in the NCBI database.

### Data availability.

The data of the raw reads and complete genome sequences of these five strains have been deposited in the SRA and GenBank (Table 1), respectively, under the BioProject accession number PRJNA693803.
